# The Impact of Health Information Privacy Concerns on Engagement and Payment Behaviors in Online Health Communities

**DOI:** 10.3389/fpsyg.2022.861903

**Published:** 2022-04-08

**Authors:** Banggang Wu, Peng Luo, Mengqiao Li, Xiao Hu

**Affiliations:** ^1^Business School, Sichuan University, Chengdu, China; ^2^School of Finance, Southwestern University of Finance and Economics, Chengdu, China

**Keywords:** privacy concerns, health information, online behavior, payment behavior, online health communities

## Abstract

Online health communities (OHCs) have enjoyed increasing popularity in recent years, especially in the context of the COVID-19 pandemic. However, several concerns have been raised regarding the privacy of users’ personal information in OHCs. Considering that OHCs are a type of data-sharing or data-driven platform, it is crucial to determine whether users’ health information privacy concerns influence their behaviors in OHCs. Thus, by conducting a survey, this study explores the impact of users’ health information privacy concerns on their engagement and payment behavior (Paid) in OHCs. The empirical results show that users’ concerns about health information privacy reduce their Paid in OHCs by negatively influencing their OHC engagement. Further analysis reveals that if users have higher benefit appraisals (i.e., perceived informational and emotional support from OHCs) and lower threat appraisals (i.e., perceived severity and vulnerability of information disclosure from OHCs), the negative effect of health information privacy concerns on users’ OHC engagement will decrease.

## Introduction

Supported by the rapid development of cutting-edge internet technologies, online health communities (OHCs) are enjoying unprecedented popularity because of the increased demand for timely and high-quality medical services (Nangsangna and Da-Costa Vroom, 2019; Wong and Cheung, 2019; Wang X. et al., 2020). Additionally, the COVID-19 pandemic may have further fostered their use due to individuals’ concerns about infection (Gong et al., 2020; Wang D. et al., 2020; Huang et al., 2021). For instance, Chunyu, one of the most popular OHCs in China, has 140 million active users and 630,000 health professionals, cumulatively serving more than 400 million patients. Moreover, OHCs provide a platform for patients to access health information, share treatment solutions, discuss medical concerns, and consult health professionals about medical issues (Yan and Tan, 2014; Zhang et al., 2018; Silver and Huang, 2020; Dahl et al., 2021). More importantly, OHCs generate enormous economic (Nambisan, 2011) and social value (Goh et al., 2016; Liu S. et al., 2020). For instance, engaging in OHCs not only provides patients with informational and social support (Mo and Coulson, 2012; Petrovčič and Petrič, 2014; Zhang et al., 2018; Agarwal et al., 2019), but also narrows the gap in the quality of medical services between urban and rural areas (Goh et al., 2016; Cao and Wang, 2018). Online health communities also allow the relationship between health professionals and patients to be improved (Liu Q. B. et al., 2020). However, some people may be reluctant to engage in OHCs due to privacy concerns, as sharing of information in OHCs is inevitably linked with the possibility of privacy violations. A survey by Auxier et al. (2020) showed that most users feel insecure about their personal information and that they have little or no control over it. In the context of health information, the consequences of data breaches are significant (Grundy et al., 2019). For instance, users may face social stigma and discrimination when buying insurance and finding jobs (Rohm and Milne, 2004; Goldfarb and Tucker, 2012; Zhang et al., 2017; Pettinico et al., 2020). Thus, this study focuses on the impact of users’ health information privacy concerns on their engagement and payment behavior (Paid) in OHCs and the moderating effects of users’ benefit appraisal (BA) and threat appraisals (TA) for information-sharing behaviors.

Although some scholars have analyzed the impact of health information privacy concern on information disclosure intention in OHCs (Zhang et al., 2017), the influence of privacy concerns on some forms of engagement needs to be explored. Therefore, we extend the existing literature by exploring the relationship between health information privacy concerns and users’ information consumption, information production, and Paid in OHCs. The extant research has analyzed the impact of information privacy concerns on users’ online engagement. As online activities play an increasingly important role in people’s everyday lives, it is widely acknowledged that privacy concerns influence users’ online behaviors (Dinev and Hart, 2005; Jang and Sung, 2021). Users may have a completely different attitude toward health records, financial information, personal identity, and eating habits (Smith et al., 2011; Gal-Or et al., 2018; Pettinico et al., 2020; Slepchuk et al., 2022), as well as varying sensitivities to privacy concerns (Jozani et al., 2020). When users have a more robust privacy control, they have increased awareness of how their data are collected and used, tend to have lower privacy concerns, and engage more frequently with online communities (Jozani et al., 2020), for example, by making more online payments for services. However, the privacy concerns on OHC are unique and important compared to other online platforms, which could be clarified from the much more serious consequences of privacy violation (e.g., discrimination and social stigma). For example, when people with infectious diseases (e.g., Tuberculosis, Hepatitis B) seek treatment advice on OHCs, they may face employment discrimination by the employer if their personal information is leaked. Also, lesbian, gay, bisexual, and transgender people may encounter discrimination when the sexual orientation they choose on OHC is exposed to their friends or family. Thus, considering the high sensitivity of health information (Kopalle and Lehmann, 2021), different motivations for engagement (Ward et al., 2005), and strict requirements for information accuracy (Zhao and Zhang, 2017), the influencing mechanism may show a different pattern, while conclusions based on other online communities should not be extended directly to OHCs. To this end, the relationship between users’ health information privacy concerns and their OHC behaviors should be explored.

The previous research has not considered the moderating effect of users’ perceived benefits and threats of sharing decision-related information on their OHC behaviors. As for the perceived benefits, people could obtain social support (e.g., informational support and social support) by participating in OHCs, where they can search for health information, receive suggestions on health issues, and obtain timely medical help in a more convenient, efficient, and effective manner (Wang X. et al., 2020), as well as reduce their stress and loneliness by talking to people in similar situations. Sharing of information is inevitably linked to privacy violations that may lead to negative consequences such as employment issues, insurance discrimination, identity theft, blackmail, targeted ad, and embarrassment (Crossler and Bélanger, 2019; Pettinico et al., 2020; Slepchuk et al., 2022), which increase users’ TA of their OHC engagement. Given that previous research suggests that people may act less rationally when they weigh the benefits and threats of OHC engagement (Barth et al., 2019; Jang and Sung, 2021), it is reasonable to assume that users’ evaluation of potential benefits and threats may moderate the relationship between users’ health information privacy concerns and their OHC behaviors.

Thus, by conducting a survey on Credamo (a platform that provides scientific questionnaire data services for scholars in more than 1,800 universities worldwide), this study explores the impact of users’ health information privacy concerns on their engagement and Paid in OHCs and the moderating effects of users’ BA and TA for information-sharing behaviors. We find that users’ health information privacy concerns have a negative relationship with their Paid in OHCs, which is mediated by users’ OHC engagement. Additionally, users’ benefit appraisals (BA) regarding informational and emotional support from OHCs positively moderate the negative relationship between their concerns about health information privacy and their OHC engagement. Further, users’ TA regarding the severity and vulnerability of information sharing in OHCs negatively moderate the effect.

## Literature Review

### Health Information Privacy Concerns and Online Engagement

Users must accept service providers’ privacy terms before being granted access to online platforms; these terms typically state that users agree to provide their private information as well as grant service providers the right to use their data (Bansal et al., 2010; Zhang et al., 2017). Nowadays, online platforms automatically track users’ behavior and constantly collect data (Dogruel et al., 2017; Wottrich et al., 2018). They tend to share said data with third parties such as businesses and the government (Acquisti et al., 2015). Inevitably, this is associated with an increased risk of privacy violations (Crossler and Bélanger, 2019; Gerhart and Koohikamali, 2019). Thus, the lack of basic security regulations on online platforms (Pour et al., 2019) fuels privacy concerns, as users’ private information may become exposed (Malm, 2018) and their anonymity could become compromised—only 15 demographic features are needed to correctly identify an individual based on online data (Rocher et al., 2019).

The extant research suggests a negative relationship between information privacy concerns and users’ engagement (UE) in online communities, which can be analyzed from two perspectives: the sensitivity of the information and users’ control over their data (Büchi et al., 2016; Jozani et al., 2020). Privacy concerns are affected by information sensitivity, whereas the level of sensitivity is determined by potential monetary, physical, social, and psychological risks caused by misuse (Milne et al., 2016; Gu et al., 2017; Kim et al., 2019). For instance, when information with a low sensitivity level (e.g., dietary habits) is collected, users may not be concerned; since the service provider may process the data comprehensively, more customized services will be provided and enhance users’ experiences (Smith et al., 2011; Gal-Or et al., 2018). However, information such as health information, financial information, shopping history, and personal identity is considered highly sensitive. The unintended disclosure of these personal data may cause significant consequences such as employment issues, insurance discrimination, identity theft, extortion, targeted ads, and embarrassment (Goldfarb and Tucker, 2012; Crossler and Bélanger, 2019; Pettinico et al., 2020; Slepchuk et al., 2022), which may reduce the level of user engagement in online communities (Jozani et al., 2020).

Furthermore, users feel that they have higher control over their data when they have more knowledge regarding how a specific data are collected and used (Büchi et al., 2016; Jozani et al., 2020). However, users seldom know the concrete procedure of the data collection and sharing process (Zarouali et al., 2018). A vivid example of this is the Facebook–Cambridge Analytica incident (Cadwalladr and Graham-Harrison, 2018). If users believe they have a higher degree of privacy control, their privacy concerns may be reduced, leading to higher engagement in online communities (Jozani et al., 2020). Accordingly, clear privacy notifications, the use of explicit permission requests and more restrictive privacy settings, and legislative actions could reduce users’ privacy concerns and motivate them to be engaged (Acquisti et al., 2015; Widjaja et al., 2019).

Privacy concerns comprise an important factor that influences UE in online communities (Dinev and Hart, 2005; Jang and Sung, 2021) as people’s lives have become increasingly entwined with online services. Thus, the potential benefits of the engagement may outweigh users’ privacy concerns when conducting a cost-benefit analysis, which may lead them to play an active role in online communities (Debatin et al., 2009). Furthermore, personal traits may serve as a moderator when users assess the privacy threats and engagement benefits (Aivazpour and Rao, 2020; Wirth et al., 2021).

### Antecedents of Engagement in Online Health Communities

People decide to be involved in OHCs by analyzing the benefits and threats of using them. The extant research has identified the antecedents of engagement, among which informational support and emotional support are the most significant (Ray et al., 2014; Gibbs et al., 2016; Yan et al., 2016; Wang et al., 2017). Informational support and emotional support, taken together, comprises social support (Chen et al., 2020), which has been regarded as the key benefit for engagement in OHCs sought by the users (Welbourne et al., 2013; Yan and Tan, 2014; Zhang et al., 2018). First, regarding the informational support, OHCs serve as platforms that connect patients in similar situations (Chou et al., 2018; Li et al., 2018). By communicating with each other, patients may obtain timely treatment solutions more conveniently and efficiently (Wang X. et al., 2020), especially considering the significant medical burden (Wu and Lu, 2018) of Chinese hospitals, where it is quite difficult to make an appointment with elite physicians (Huang et al., 2021). Moreover, searching for an information in OHCs is cost-effective, which may increase the possibility that patients use OHCs (Gao et al., 2021), since the high costs of medical treatment may worsen patients’ physical health due to stress (Gao et al., 2021). Second, emotional support is also important for patients (Yan and Tan, 2014; Gibbs et al., 2016), who require sympathy, encouragement, companionship, and other kinds of emotional support (Jin et al., 2016). The OHCs may serve as a source of emotional support by allowing users to communicate with each other (Yu et al., 2015). Compared with health professionals’ suggestions—which are more direct due to doctors’ tight schedules (Wang L. et al., 2020)—exchanges in OHCs are more emotionally supportive (Van Oerle et al., 2016) and can reduce patients’ stress (Rains and Young, 2009; Goldsmith et al., 2013). Third, regarding additional factors, psychological benefits like individual reputation, self-esteem, and self-efficacy also facilitate people’s engagement with OHCs (Van Uden-Kraan et al., 2009; Bansal et al., 2010; Mo and Coulson, 2012; Petrovčič and Petrič, 2014). Moreover, by providing suggestions and helping others, users may obtain more accurate medical knowledge and increase their health literacy, eventually developing non-professional expertise (Nettleton et al., 2005; Griffiths et al., 2012; Chen et al., 2019). The safety concerns related to the COVID-19 pandemic provide another reason for the usage of online medical services (Gong et al., 2020; Huang et al., 2021), as nearly 41% of cases are associated with hospital visits (Wang D. et al., 2020).

Certain risks may reduce the benefits from engagement in OHCs and impair people’s eagerness to participate. The previous research has mainly focused on concerns regarding health misinformation. Additionally, oversimplification (Zhao et al., 2021), lack of scientific evidence (Chou et al., 2018), and deliberate distortion (Waszak et al., 2018) may generate misinformation. The lack of gatekeepers causes misinformation to spread quickly (Bode and Vraga, 2017; Li et al., 2022). Additionally, people who lack health and scientific knowledge may be misled *via* the echo chamber effect (Brady et al., 2017; Vogel, 2017). Therefore, for health issues, the accuracy of the information is important because misinformation may lead to poor health outcomes or even death (Zhao and Zhang, 2017). Thus, using OHCs carries significant risks (Chen et al., 2018). Although some cutting-edge technologies, such as deep learning and machine learning (Sicilia et al., 2018; Kumar et al., 2019; Ruokolainen and Widén, 2020; Song et al., 2021) are used to develop a filtering system for misinformation; however, these methods still face many challenges and cannot fully eliminate risks (Li et al., 2022). Thus, people should exercise caution when seeking advice through OHCs to avoid misinformation (Pluye et al., 2019).

Health professionals are significant participants in OHCs and their decision process for engagement is also worth investigating in order to have a comprehensive view of the antecedents of engagement in OHCs. Three main drivers can be identified in prior literature on engagement in OHCs (Zhou et al., 2019). First, there is technical competence, which refers to the ability to provide patients with professional services (Robin DiMatteo and Hays, 1980). Many researchers have found a positive relationship between health professionals’ technical competence and OHC engagement (Zhou et al., 2018, 2019; Li et al., 2019). Online services may also improve health professionals’ performance in hospitals, including operational efficiency, resource utilization efficiency, and patient satisfaction, which may, in turn, enhance their technical competence and satisfy their self-efficacy (Wu et al., 2021). Second, regarding online reputation and economic rewards, positive relationships have been identified among engagement in OHCs, online reputation, and economic rewards (Zhang et al., 2017). For health professionals, having a good reputation not only helps them improve their self-efficacy and achieve internal satisfaction (Constant et al., 1996) but also brings potential economic benefits. In the context of information asymmetry (Yan et al., 2016), wherein patients choose their physicians, they often use their online reputation as reference (Deng et al., 2019). A higher online reputation is associated with more appointments (Liu et al., 2016). In addition, more votes and thumbs will be received with an increasing number of views of their homepages (Luo et al., 2017; Deng et al., 2019; Li et al., 2019). Therefore, health professionals are motivated to provide services in OHCs, which will, in turn, gradually help them build their brand (Lu and Wu, 2016), attract more patients, and increase sales (Li et al., 2019), eventually resulting in economic and social benefits (Guo et al., 2017).

Nevertheless, two factors may prevent health professionals from participating in OHCs. First, as specialists, health professionals must ensure that their answers are accurate, professional, and precise, compared with normal users (Hennig-Schmidt and Wiesen, 2014), since even a small mistake may incur tremendous criticism that potentially damages their reputation. In other words, health professionals pay a high price when helping others in OHCs (Zhang et al., 2017). Second, health professionals in China are required to carry out scientific research outside of their working hours. Thus, resource constraints (e.g., limited time and energy) may prevent them from continuing their online activities (Wang L. et al., 2020).

### Summary

After reviewing relevant literature comprehensively, to the best of our knowledge, the research gap could be concluded into the following three aspects. First, few studies have been conducted on the impact of information privacy concerns on users’ OHC engagement. Second, previous research has mainly discussed UE in OHCs, whereas Paid remains relatively unexplored. Third, although many scholars have suggested that people may become less rational when carrying out benefit-threat analysis, BA and TA within the context of OHCs are not clarified.

To fill in these research gaps, this study takes health information privacy concerns into the antecedents’ analysis of UE in OHCs, and sheds light on users’ Paid in OHCs, while also incorporates BA and TA within the context of OHCs. All the results and conclusions are based on and summarized from a well-designed survey that is conducted on a professional scientific data services platform, Credamo.

## Hypothesis Development

### Health Information Privacy Concerns and User Engagement in Online Health Communities

Users make their engagement decisions by evaluating their potential threats and benefits. To assess the potential benefits of informational and emotional support, users must accept OHCs’ privacy terms and finish the form containing their health information. Unlike other online communities (Kopalle and Lehmann, 2021), information in OHCs has two main features: higher accuracy and higher sensitivity (Markos et al., 2018). First, users cannot falsify or withhold their health information due to privacy concerns because less accurate health information could lead to wrong treatment solutions or even death (Crié and Chebat, 2013). For example, if a person is allergic to a particular drug and does not inform the doctor, the doctor uses the drug and surely the effect is counterproductive. Second, the higher sensitivity of the information contained in OHCs may lead to data breaches, causing significant problems such as discrimination and annoying advertisements. Huge commercial interests motivate privacy violations. For example, in many families with newborns, advertisements for various products and services have been accurately sent to phones and mailboxes of young parents. Employers also want to buy health private data to get real health status of job applicants. Although some regulations and laws on information use have been introduced to the public (Kopalle and Lehmann, 2021), the effects of these policies remain to be seen.

In summary, if the users are prepared to engage in OHCs, it is important to ensure the accuracy of the information they disclose. Given the potential threat of information disclosure and privacy concerns, the foreseeable benefits from engagement may be hedged. Thus, we propose Hypothesis 1 as follows:

*Hypothesis 1* (H1): Health information privacy concerns negatively influence OHC engagement.

### User Engagement and Payment Decision for Services in Online Health Communities

On sharing their experiences with others, users can establish a deep and meaningful connections, which in turn enhances trust and communication among users; thus, users eventually gain informational and emotional support *via* OHCs. Users develop a sense of engagement and belongingness when they frequently share and seek information online (Wellman and Gulia, 1999; Bateman et al., 2011). The more frequently they use OHCs, the easier it is for users to get emotionally attached to OHCs (Mirzaei and Esmaeilzadeh, 2021). Furthermore, the users tend to feel ashamed and guilty if they simply access information and emotional support without providing the same to others (Mirzaei and Esmaeilzadeh, 2021). Only when the users strike a balance between providing and receiving support can they develop a sense of belongingness, which they subsequently become afraid of losing (Guo et al., 2016; Gao et al., 2017). Therefore, most users tend to make contributions to OHCs (Bowden et al., 2018; Liu S. et al., 2020), eventually reaching a higher level of engagement (Mirzaei and Esmaeilzadeh, 2021). Obviously, the Paid is a form of higher-level engagement. For example, you will never transfer money to strangers, and will never buy services from unknown providers due to quality concerns.

Moreover, research on other online communities has shown that users are more inclined to pay for online services in order to gain stable socializing functions and entertainment value from online communities after they have become emotionally attached to said communities (Oestreicher-Singer and Zalmanson, 2013; Brettel et al., 2015; Meng et al., 2020). Thus, we develop the following hypothesis:

*Hypothesis 2* (H2): OHC engagement positively influences users’ Paid in OHCs.

### The Mediating Effect of User Online Health Communities Engagement

The previous research has suggested that when users frequently engage in online communities, they may gain a sense of belongingness, which in turn increases the possibility that they pay for online services (Brettel et al., 2015; Meng et al., 2020). Thus, we can regard Paid as a deeper level of engagement. Based on H1 and H2, it is reasonable to assume that health privacy information concerns (PICs) may influence Paid in OHCs. Intuitively speaking, lower-level engagement should serve as a transitional phase for deeper engagement (e.g., Paid). For instance, you won’t know how to run unless you know how to walk. Therefore, it is reasonable to postulate that OHC engagement plays a mediating role in the relationship between users’ health information privacy concerns and their Paid in OHCs. To this end, we hypothesize the following:

*Hypothesis 3* (H3): Health information privacy concerns have a negative relationship with users’ Paid in OHCs, mediated by their OHC engagement.

### The Moderating Effects of Benefit and Threat Appraisals

On engaging in online communities, the users can obtain multiple benefits, such as increased convenience in daily life activities, a sense of self-value, and enjoyment (Heravi et al., 2018). These merits may influence users’ degree of engagement, which could moderate the relationship between health information privacy concerns and OHC engagement. The users may have different motivations for using OHCs, where seeking social support (i.e., informational and emotional support) is the priority instead of monetary rewards (Mirzaei and Esmaeilzadeh, 2021). This is easy to understand since money can never buy health. Informational support refers to the possibility for OHC users to search for health information, suggestions on health issues, and timely medical help in a convenient, efficient, and effective manner (Wang X. et al., 2020). Emotional support refers to the possibility for OHC users to gain valuable insights from individuals in similar situations, which could reduce their stress and loneliness, cultivate a positive attitude, and help them develop self-value and self-efficacy (Gibbs et al., 2016). Although deeper privacy concerns may reduce users’ willingness to share their sensitive health information (Dinev and Hart, 2005) and may lead to a lower degree of engagement, an individual’s evaluation of potential benefits could have a significant moderating effect on this negative relationship. The previous research has suggested that people may act less rationally when carrying out a benefit–threat analysis (Barth et al., 2019; Jang and Sung, 2021) and often overestimate benefits (Manago and Melton, 2019). For example, when we are so enthusiastic for a new dress, we may only see how good the dress is, ignoring the fact that we may already have a lot of them in similar style, which leads to irrational purchase. This lack of rationality paves the way for analyzing the moderating effect from the perspective of users’ BA. Specifically, users who perceive the benefits of information-sharing and emotional support in OHCs to be higher tend to have lower privacy concerns regarding OHC engagement. Thus, we propose the following hypothesis:

*Hypothesis 4a* (H4a): Users’ BA (i.e., perceived benefits of informational and emotional support in OHCs) increase the likelihood that users with high health information privacy concerns will engage in OHCs.

Threat appraisals (which negatively moderate the relationship between health information privacy concerns and OHC engagement) comprise two dimensions: perceived severity and perceived vulnerability of information disclosure. The former relates to the seriousness of the consequences of a potential data misuse, as evaluated by the user; the latter refers to the self-evaluation of the possibility of data misuse (Rogers, 1975; Zhang et al., 2017). First, a higher perceived severity means greater expected seriousness. Privacy violations may result in negative consequences like discrimination as well as social stigma (Crossler and Bélanger, 2019; Pettinico et al., 2020; Slepchuk et al., 2022), some of which can be severe. Users tend to adjust their level of engagement based on the perceived severity of potential privacy violations (Zhang et al., 2017; Barth et al., 2019). They are inclined to be more prudential when perceived severity remains at a higher level (Dinev and Hart, 2005; Herath et al., 2014). In other words, people may be less involved in OHC when the consequences are deemed too severe to bear. Second, a higher perceived vulnerability indicates that users believe they are more prone to privacy violations—that is, that their data are likely to be misused. Accordingly, users will take protective measures to reduce the expected threat and become less active in OHCs (Liu et al., 2017; Barth et al., 2019). Thus, it is reasonable to assume that both perceived severity and vulnerability (which comprise OHC users’ TA) amplify the negative relationship between the health information privacy concerns and the users’ OHC engagement. In other words, the users who have greater expected seriousness or believe they are more prone to privacy violations will be less active in OHCs. Thus, we posit the following hypothesis:

*Hypothesis 4b* (H4b): Users’ TA (i.e., perceived severity and vulnerability of information disclosure in OHCs) decrease the likelihood that users with significant health information privacy concerns will engage in OHCs.

**FIGURE 1:**
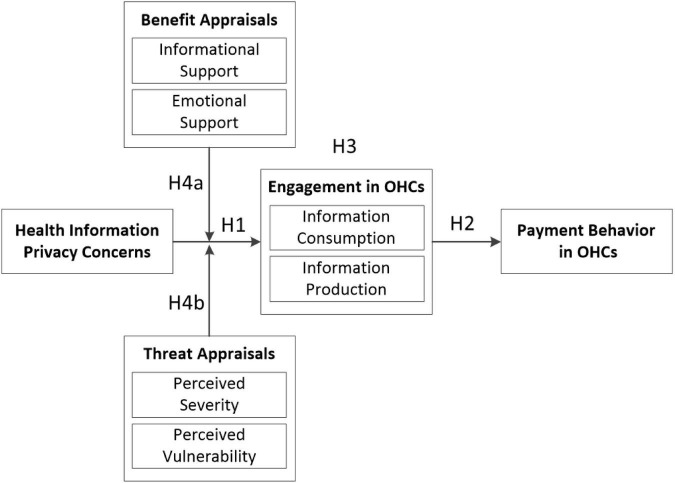
Theoretical model.

Figure 1 shows the theoretical model of this study based on its theoretical framework and research hypotheses.

## Research Design

### Data Source

For this study, we designed a questionnaire and uploaded it to the online platform, Credamo. Credamo is a data platform committed to providing solutions for large-scale research, data collection, modeling analysis, and commercial applications for scientific research institutions, enterprises, and individuals. Credamo provides scientific research and educational data services for teachers and students in more than 1,800 universities worldwide. The scope of services covers various disciplines such as management, psychology, medicine, sociology, tourism, and hotel management, while its research studies are accepted by top academic journals. Credamo also provides large-scale research, government affairs management, human resource management, consumer product development, and other information services for both governments and enterprises.

Note that all the data for the present study were collected from Credamo, where data representativeness is guaranteed because of Credamo’s unique sampling method and comprehensive register of users, which covers all provinces, cities, and regions in China and most of their occupations. Table 1 presents the sample statistics.

**TABLE 1:** Sample characteristics (*N* = 480).

Variable		Count	Percentage (%)
Health conditions (HC)	Very poor	14	2.91
	Poor	130	27.03
	Fair	267	55.72
	Good	69	14.35
Age	<20	37	7.71
	20–29	241	50.21
	30–39	156	32.5
	40–49	32	6.67
	≥50	14	2.92
Education	Primary school or below (=1)	1	0.21
	Junior high school (=2)	3	0.62
	High school (=3)	22	4.57
	College Degree (=4)	52	10.81
	Bachelor degree (=5)	349	72.77
	Master degree (=6)	51	10.60
	Ph.D degree (=7)	2	0.42
Gender	Male (=1)	192	40
	Female (=0)	288	60

The questionnaire design process was divided into two stages. First, we sent the questionnaire to experts, who made suggestions on its design. After receiving their feedback, we optimized and modified the questionnaire and developed a final version. Second, we uploaded the questionnaire to Credamo and collected users’ answers. Specifically, the final version of the questionnaire comprised four parts. The first section details the purpose of the questionnaire, emphasizing that data collection is purely conducted for academic purposes, and guarantees the absolute confidentiality of the information. Then, we confirm whether the applicant has previously used OHCs. If not, the survey ends directly, which allows us to ensure the reliability of the results. Next comes the core section, collects data on UE in OHCs, including “views on filling in personal health information,” “views on personal health information leakage,” “specific usage behaviors,” and “the possibility of paying for online medical consulting.” Finally, we asked the participants to fill in their demographic information such as gender, education level, and health status. The constructs and corresponding items are outlined in the first two columns of Table 2.

**TABLE 2:** Research constructs, measurements, item loadings, and validities.

Construct	Item	Standard Loading	AVE	CR	Cronbach’s Alpha
PIC (privacy information concern)	(1) I feel that it is not advisable to fill in personal health information in the online health community	0.847	0.714	0.882	0.853
	(2) Once the personal health information in the online health community is filled in, it will be abused by companies	0.840			
	(3) Once the personal health information in the online health community is filled in, it will be shared by the company or sold to others	0.848			
BA (benefit appraisals)	(1) When I need help in the online health community, someone will give me advice	0.851	0.736	0.916	0.871
	(2) When I encounter difficulties, users in the online health community will help me find the reasons and provide suggestions	0.846			
	(3) When I encounter difficulties, users in the online health community will comfort and encourage me	0.859			
	(4) When I encounter difficulties, users in the online health community will express their concern for me	0.863			
TA (threat appraisals)	(1) Personal health information in the online health community is at risk of being shared or sold	0.850	0.724	0.887	0.913
	(2) My personal health information in the online health community may be shared or sold	0.852			
	(3) Once I fill in my personal health information in the online health community, my information may be shared or sold	0.851			
UE (user engagement)	(1) I will share my treatment process in the online health community	0.870	0.710	0.907	0.755
	(2) I will make comments on the doctor in the online health community	0.793			
	(3) I will “like” other users’ contents in the online health community	0.893			
	(4) I will recommend the online health community to my friends	0.711			
PB (payment behavior)	Have you ever paid to doctors for medical consultations in the online health community?				

To accurately identify the impact of privacy concerns on users’ participation in OHCs, the model incorporates many control variables. Considering that factors such as age, educational level, gender, and health status may impact the relationship between privacy concerns and OHC engagement, we incorporated them into the model as control variables. The introduced control variables were consistent with those in the existing literature.

### Measurement Assessment

Before identifying the structural model and testing for mediating and moderating effects, several tests were carried out to ensure the validity of the data. First, the standardized loadings of each item on the construct were computed, as shown in the third column of Table 2. We may safely draw the conclusion that the item reliability of all measures is good because all standard loadings are greater than 0.7 (Fornell and Larcker, 1981). Second, the average variance extracted values were calculated, and all four values were greater than 0.5 (Fornell and Larcker, 1981). Third, we derived composite reliability and Cronbach’s alpha. All the values are greater than 0.7 (Hair et al., 2011), indicating the internal consistency and reliability of the measures. Combining the results of the four tests mentioned (see Table 2), we can conclude the validity of the data.

## Empirical Results

### Correlation

To exclude the influence of multicollinearity, we derived the correlation matrix; the results are shown in Table 3. Note that we only show the correlation coefficients. Multicollinearity exists only when the coefficient is greater than 0.5 and is statistically significant. Table 3 shows that no coefficients satisfied this condition, indicating that no multicollinearity effects exist in the model. Additionally, consistent with the intuitive negative relationship between UE and their Paid, the correlation coefficient between the UE and Paid is positive. Variance inflation factors (VIFs) were calculated to test for other potential multicollinearity problems. The maximum VIF obtained in any of the models was 4.23 and the mean VIF of the complete model [i.e., Model (4) in Table 4] was 3.38, which was substantially below the rule-of-thumb cutoff of 10 for regression models (Ryan, 1997). Therefore, multicollinearity was not considered an important issue in these analyses.

**TABLE 3:** Correlation matrix.

Variables		1	2	3	4	5	6	7	8
1	Paid user								
2	UE	0.411*							
3	PIC	−0.274*	−0.369*						
4	BA	−0.131*	−0.06	0.301*					
5	TA	−0.260*	−0.334*	0.703*	0.452*				
6	HC	−0.08	0.04	−0.01	0.00	0.00			
7	Gen	0.115*	0.146*	−0.06	−0.06	−0.092*	0.03		
8	Age	0.116*	0.145*	−0.01	−0.06	−0.091*	−0.106*	0.096*	
9	Degree	−0.04	0.02	0.05	0.04	0.124*	0.093*	−0.03	−0.187*

*p < 0.05.

**TABLE 4:** Results of the moderating effects.

	UE			
	(1)	(2)	(3)	(4)
PIC	−0.199***	−0.486***	−0.524***	−0.652***
	(0.042)	(0.136)	(0.137)	(0.163)
PIC × BA		0.072**		0.075**
		(0.032)		(0.035)
PIC × TA			−0.085**	−0.066**
			(0.034)	(0.032)
BA	0.068**	−0.094	0.077**	−0.037
	(0.034)	(0.081)	(0.034)	(0.086)
TA	−0.114**	−0.100**	−0.288***	−0.241**
	(0.039)	(0.039)	(0.080)	(0.086)
HC = Poor	0.157	0.189	0.186	0.202
	(0.183)	(0.182)	(0.182)	(0.182)
HC = Fair	−0.183	0.209	−0.322	−0.789
	(0.719)	(0.178)	(0.727)	(0.819)
HC = Good	0.135	0.170	0.164	0.182
	(0.188)	(0.188)	(0.188)	(0.188)
Gen = Male	0.179**	0.168**	0.174**	0.167**
	(0.059)	(0.059)	(0.059)	(0.059)
Age	0.101**	0.097**	0.097**	0.095**
	(0.035)	(0.035)	(0.035)	(0.035)
Education fixed effect	Control	Control	Control	Control
City fixed effect	Control	Control	Control	Control
Constant	0.862	0.805	1.282**	1.456**
	(0.629)	(0.643)	(0.647)	(0.658)
N	480	480	480	480
Adjusted *R*^2^	0.215	0.222	0.224	0.226

Standard errors in parentheses; **p < 0.01, ***p < 0.001.

### Main Effect

This study employs the ordinary least squares method (OLS) to test the relationship between users’ health information privacy concerns and their engagement in OHCs. The results are shown in Models (1) and (2) of Table 5. Model (1) only incorporates the control variables. Model (2) adds the dependent variable PIC and shows that the effect of PIC on UE is significantly negative (β = −0.199, *p* < 0.001). Therefore, H1 (health information privacy concern negatively influences UE in OHCs) is supported.

**TABLE 5:** Results of the main and mediating effects.

	UE		Paid			
	OLS		Logit model			
	(1)	(2)	(3)	(4)	(5)	(6)
PIC		−0.199***			−0.452**	−0.241
		(0.042)			(0.158)	(0.172)
UE				1.316***		1.267***
				(0.191)		(0.194)
BA	−0.083	0.068**	0.074**	−0.250	−0.095	−0.250
	(0.154)	(0.034)	(0.035)	(0.173)	(0.156)	(0.173)
TA	−0.610***	−0.114**	−0.238***	−0.223	−0.325**	−0.223
	(0.130)	(0.039)	(0.030)	(0.178)	(0.165)	(0.178)
HC = Poor	−0.148	−0.271	0.191	−0.565	0.157	−0.590
	(0.743)	(0.750)	(0.187)	(0.841)	(0.183)	(0.843)
HC = Fair	0.209	0.237	0.249	−0.752	0.238	0.251
	(0.178)	(0.177)	(0.181)	(0.818)	(0.177)	(0.177)
HC = Good	−0.769	0.135	0.162	−1.249	−0.887	−1.284
	(0.752)	(0.188)	(0.193)	(0.852)	(0.760)	(0.854)
Gen = Male	0.499**	0.179**	0.177**	0.287	0.532**	0.319
	(0.245)	(0.059)	(0.060)	(0.265)	(0.249)	(0.267)
Age	0.271*	0.101**	0.090**	0.131	0.309**	0.147
	(0.147)	(0.035)	(0.036)	(0.151)	(0.148)	(0.152)
Education fixed effect	Control	Control	Control	Control	Control	Control
City fixed effect	Control	Control	Control	Control	Control	Control
Constant	1.590	0.862	(0.643)	−2.640	1.688	−2.421
	(1.762)	(0.629)	480	(1.930)	(1.759)	(1.931)
*N*	480	480	480	480	480	480
Adjusted *R*^2^	0.095	0.215				
Pseudo *R*^2^			0.179	0.198	0.111	0.203

Standard errors in parentheses; *p < 0.05, **p < 0.01, ***p < 0.001.

Since Paid is a dichotomous variable, we use a logit model to test the relationship between user OHC engagement and Paid. The results are shown in Models (3) and (4). Model (3) is the baseline. Model (4) shows that the coefficient of UE is positively significant (β = 1.316, *p* < 0.001). Therefore, H2 (OHCs engagement positively influences users’ Paid in OHCs) is supported.

The coefficients of the control variables show that a higher degree of engagement exists for users who are male and older.

### Mediating Effect

We refer to the method of Baron and Kenny (1986) and conduct three-step econometric models to test the mediating model. The first step examines whether health information privacy concerns have an impact on user OHC engagement (i.e., PIC → UE). The results in Model (2) of Table 5 support this relationship. The second step examines whether UE in OHCs has an impact on their Paid (i.e., UE → Paid). The results in Model (4) of Table 5 support this.

Based on these results, the third step examines the relationship between health information privacy concerns and users’ Paid (i.e., PIC → Paid). Model (5) shows that the effect of PIC on Paid is significantly negative (β = -0.452, *p* < 0.01). After incorporating the mediating variable UE in Model (5), the coefficient of UE is still significant at the significance level of 0.001, while the marginal effect of UE on Paid changes from -0.452 to -0.241, and becomes insignificant (*p* > 0.05). The results indicate that user OHC engagement plays a fully mediating role in the impact of health information privacy concerns on users’ Paid, supporting H3.

### Moderating Effects

To test the moderating effect of BA and TA on the relationship between users’ health information privacy concerns and their OHC engagement, we add the interaction term between health information privacy concerns and BA (i.e., PIC × BA), as well as the interaction term between PIC and TA (i.e., PIC × TA) into the regression model. The results are shown in Table 4.

Models (2) and (4) show that the impact of PIC × BA on UE is positive at the significance level of 0.01, indicating that users’ BA positively moderate the relationship between health information privacy concerns and users’ OHCs engagement. Thus, H4a is supported. In addition, the coefficient of PIC × TA is negative at the significance level of 0.01, which supports H4b that users’ TA increase the likelihood for users with significant health information privacy concerns to engage in OHCs.

**FIGURE 2:**
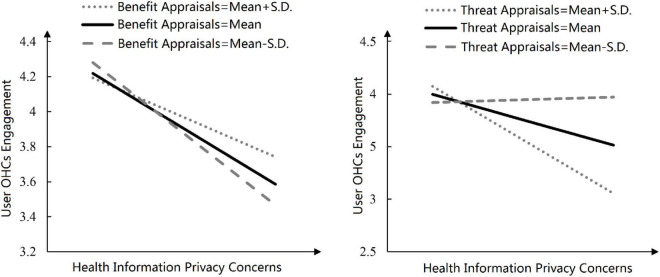
Interaction graphs.

Figure 2 shows the moderating effects more intuitively. We added and subtracted a standard deviation on BA and TA and further drew on the relationship between PIC and UE at the different levels of BA or TA. The analysis logic of the two graphs is similar, and both focus on the steepness of the line. As shown on the left side of Figure 2, when BA changed from BA – SD to BA + SD, the line becomes flattener, and the absolute value of the slope decreased, indicating that users’ BA positively moderate the negative relationship between users’ health information privacy concerns and their OHCs engagement. The right side of Figure 2 shows that when TA changed from TA − SD to TA + SD, the line becomes steeper, indicating that users’ TA negatively moderate the relationship between users’ health information privacy concerns and their OHC engagement.

## Discussion and Conclusion

### Conclusion

This study explores the impact of users’ health information privacy concerns on their engagement and Paid in OHCs, as well as the moderating effects of users’ benefits and TA of sharing information. There are three main conclusions. First, we indicate a negative relationship between users’ health information privacy concerns and their OHC engagement. The higher users’ health information privacy concerns, the lower their OHC engagement. Second, after analyzing Paid in the context of OHCs, we find that UE in OHCs mediates the relationship between users’ health information privacy concerns and their Paid in OHCs. That is, health information privacy concerns affect users’ Paid by influencing their OHC engagement. Third, we identify the moderating effects of BA and TA on the negative relationship between health information privacy concerns and user OHC engagement. Benefit appraisals of using OHCs increase the likelihood that individuals with high health information privacy concerns would engage in OHCs, while TA will decrease the likelihood that individuals with high health information privacy concerns would engage in OHCs.

### Theoretical Contributions

Our study’s theoretical contributions can be summarized as follows. First, it extends the existing literature on the antecedents of UE in OHCs by incorporating health information privacy concerns into its analysis. Extant research has identified the antecedents of OHC engagement, and to gain informational support, as well as emotional support, are considered the primary motivations (Ray et al., 2014; Gibbs et al., 2016; Yan et al., 2016; Wang et al., 2017). Safety concerns related to the ongoing COVID-19 pandemic have also fostered enthusiasm for engagement in OHCs (Huang et al., 2021). However, to the best of our knowledge, few studies have been conducted on the impact of information privacy concerns on user engagement. Actually, health information is highly sensitive and requires high accuracy (Markos et al., 2018), which is significantly different from the information in other online communities (Kopalle and Lehmann, 2021). Thus, conclusions in other online communities cannot be applied directly in the context of OHCs. Considering the severe consequences of data breaches, the importance of privacy concerns should not be ignored in users’ decisions to participate in OHCs.

Second, this study sheds light on users’ Paid in OHCs. On the one hand, previous research has mainly discussed UE in OHCs, whereas Paid remains relatively unexplored. OHC users primarily search for health-related information (Wang X. et al., 2020) and professional online medical services (Huang et al., 2021), while aiming to communicate with individuals who have similar experiences (Chou et al., 2018). On the other hand, the scarce studies on Paid in online communities have not been carried out within the context of OHCs. Research on other online communities has shown that users are more inclined to pay for online services to gain stable socializing functions and entertainment value from online communities after they have become emotionally attached (Oestreicher-Singer and Zalmanson, 2013; Brettel et al., 2015; Meng et al., 2020). Thus, combining health information privacy concern and online Paid could provide valuable insights.

Third, this study deals with a research gap by incorporating BA and TA within the context of OHCs. The previous research has suggested that people may become less rational when carrying out benefit–threat analysis (Barth et al., 2019; Jang and Sung, 2021), in which they are inclined to overvalue the merits (Manago and Melton, 2019). The lack of rationality paves the way for analyzing the moderating effect from the perspective of users’ benefits and TA. In contrast, users tend to be more prudential (Pluye et al., 2019) and may take protective measures to mitigate the expected threat by becoming less active in OHCs (Liu et al., 2017; Barth et al., 2019). Thus, elucidating the effects of benefit and threat appraisal is of great significance to understand OHC engagement.

### Practical Implications

The practical implications of this study can be summarized as follows. For OHC administrators, at least two objectives are elucidated in this study. First, they should aim to guarantee the protection of users’ private information. This study shows that the more concerned users are about their health information privacy, the lower their engagement in OHCs. Thus, OHC administrators should reduce users’ privacy concerns to foster the long-term development of OHCs, since OHCs are information sharing or information-driven platforms. Specifically, administrators could provide users with more options for information disclosure and increase transparency by informing users why and how their information will be used. Second, administrators should improve users’ stickiness and cultivate their sense of belonging. This study suggests that health information privacy concerns affect users’ Paid *via* their engagement in OHCs. Intuitively, if a higher proportion of users is willing to pay for services, the platform will reap higher profits; thus, OHC administrators should introduce more measures to increase UE and gradually cultivate their loyalty and sense of belongingness to the platform, which will, in turn, increase the possibility that they will pay for online services (Brettel et al., 2015; Meng et al., 2020).

Additionally, there is one meaningful implication for users. This study indicates that people who perceive the benefits of informational and emotional support from OHCs to be higher tend to engage more frequently in OHCs. In contrast, individuals who perceive the threats inherent to information disclosure to be higher reduce their OHC engagement. The previous research has suggested that people may act less rationally when carrying out benefit-threat analyses (Barth et al., 2019; Jang and Sung, 2021). Thus, users should act rationally and not overestimate or underestimate the perceived benefits and threats, thereby developing reasonable expectations for the support provided in OHCs, which could allow them to obtain a better user experience.

### Limitations and Further Research

This study has some limitations that can also provide promising directions for future research. First, the effect of differences in medical service quality should be considered, as the unbalanced distribution of medical resources caused by geographical dispersion influences UE in OHCs. Although the questionnaire designed in this study collected users’ geographic information, it did not analyze these data *vis-à-vis* the distribution of medical resources or its heterogeneity effect. China has a vast territory, and the distribution of medical resources is highly uneven. The previous studies have shown that OHCs could narrow the quality gap of medical services between urban and rural areas (Goh et al., 2016; Cao and Wang, 2018). Thus, it would be valuable to conduct more research on how medical conditions influence the decision of users with high health information privacy concerns regarding OHC engagement.

Second, the effect of the size of OHCs. Larger communities have more stable cash flows and, in turn, are less likely to suffer data breaches. Additionally, they often have more advanced technology to protect users’ data from being hacked. Thus, OHCs’ size may moderate the relationship between PICs and user engagement, which requires further exploration in future studies.

## Data Availability Statement

The original contributions presented in the study are included in the article/supplementary material, further inquiries can be directed to the corresponding author/s.

## Ethics Statement

Ethical review and approval was not required for the study on human participants in accordance with the local legislation and institutional requirements. Written informed consent from the (patients/participants or patients/participants legal guardian/next of kin) was not required to participate in this study in accordance with the national legislation and the institutional requirements.

## Author Contributions

BW designed the research framework. PL carried out the survey. ML and XH analyzed the data. BW, PL, XH, and ML wrote the manuscript. BW and PL contributed equally to the manuscript. All authors contributed to the article and approved the submitted version.

## Conflict of Interest

The authors declare that the research was conducted in the absence of any commercial or financial relationships that could be construed as a potential conflict of interest.

## Publisher’s Note

All claims expressed in this article are solely those of the authors and do not necessarily represent those of their affiliated organizations, or those of the publisher, the editors and the reviewers. Any product that may be evaluated in this article, or claim that may be made by its manufacturer, is not guaranteed or endorsed by the publisher.
